# Semi-annual Meeting North Texas Medical Association

**Published:** 1886-07

**Authors:** F. D. Thomson, Geo. R. Clayton

**Affiliations:** President; Secretary pro tem


### SOCIETY NOTES.

### SEMI-ANNUAL MEETING OF THE "NORTH TEXAS MEDICAL ASSOCIATION."

HELD IN SHERMAN, JUNE 8TH, 9TH, 10TH.

*June* 8:—The Association was called to order at 10 a. m., by F. D. Thompson, President. Geo. R. Clayton was appointed Secretary *pro tem.* The following members were present, viz:

Hooks, of Paris; Saunders and Dabney, of Bonham; Duncan, of Savoy; Moore, Van Alstyne; King, Bells; Cox, Arkada; Gilcreest, Howeth, Gainesville; Gardner, Denison; Wright, Deport; Moody, Blossom Prairie; Thompson, Stinson, Wright, Scott, Freeman, Brocket, Lankford, Nisbet, Gunby, Clayton, Sherman.

All local and visiting physicians, not members, were invited to a seat, and to participate in the proceedings during the session.

The morning session was devoted to appointing necessary committees and admitting new members.

AFTERNOON SESSION.

Met at 2 p. m. President Thompson in the chair. The section on Surgery was opened. Subject: "Surgery of the Rectum." Papers were read by Dr. Wilson, of Austin, and Stinson, of Sherman.

Papers were also read by Wright, of Deport, and Stinson, of Sherman, on "The Importance of Circumcission." The papers elicited considerable discussion—almost all the members taking part and giving their views on the subject, before the house adjourned to meet to-morrow morning at 8 o'clock.

*June* 9:—Met at 8 a m. President Thompson in the chair. The section on Gynecology was opened, the subject being, "Uterine Displacements." Papers were read by Hooks, of Paris, and Nesbit, of Sherman. Considerable discussion followed. The main point of issue being the practicability of

Uterine *Pessaries;* the Association was about divided on the subject.

The section on Surgery was reopened, and a paper, read by Dr. Goodwin, of Pottsboro, on "Fracture of the Skull, with Compression."

AFTERNOON SESSION.

Met at 2:30 p. m. Section on Practice was opened, the subject being "Bowel Troubles of Children During Dentition." Papers were read by Bacon Sanders, of Bonham, and John O. Scott, of Sherman. Considerable discussion followed.

Dr. Bucket read the report of a cure of "Cancer of the Stomach," and Dr. Thompson gave report of "Autopsy" of the same.

The Annual Address was delivered by the President during the evening, after which the Association adjourned to Steadman's Parlors, where they partook of a "banquet" given by Grayson County Medical Society and local physicians.

*June* 10: —Association met at 9 a. m. President Thompson in the chair. This session was devoted to attending to business pertaining to the Association and the appointing of committees of the various sections. The following committees were appointed, viz:

Surgery—"Dislocations":

J. M. Fort, Paris.

I. P. Gunly, Sherman;

T. M. Taylor, Sherman.

Gynæcology—"Uterine Tumors, and the Management of the Second Stage of Labor":

J. T. Wilson, Austin;

J. Duncan, Savoy;

V. A. Harveth, Gainesville.

Practice—"Pernicious Malarial Fever, as seen in this section of the country:

E. W. Rush, Paris.

J. R. Bristow, Farmington.

S. E. Bramlett. Paris.

The following gentlemen were elected to membership during the session, viz:

T. M. Taylor, Sherman.
J. W. Jackson, Sherman.
S. D. Howser, Aubrey.
Y. D. Emerson. Cameron.
I. D. Devine, White Mound.
D. Garden, Pottsboro.
J. R. Burton, Farmington.
E. W. Arnold, Whitesboro.

The Association adjourned to meet in Paris the second Tuesday in December, 18[illegible]6.

F. D. Thomson, President.

Geo. R. Clayton, Secretary *pro tem.*

The "North Texas Medical Association" has about 100 members. The territory embraces all the northern tier of country from Texarkana to the western border of Cook county. The meetings are held semi-annually, in June and December, at Sherman and Paris respectively. The meetings are generally largely attended, and the papers are on practical subjects and are generally very interesting and elicit thorough discussion of the subjects.

